# Applying a New Model for Sharing Population Health Data to National Syndromic Influenza Surveillance: DiSTRIBuTE Project Proof of Concept, 2006 to 2009

**DOI:** 10.1371/currents.RRN1251

**Published:** 2011-09-12

**Authors:** Donald R Olson, Marc Paladini, William B Lober, David L Buckeridge, ISDS Distribute Working Group, Marc Paladini, Richard T. Heffernan, Atar Baer, Michael A. Coletta, Karl Soetebier, Erin L. Murray, Lana Deyneka, Amy Ising, Ryan Gentry, Felicia Alvarez, Melissa Dimond, Bryant Thomas Karras, Kieran Moore, Ian Painter, William B. Lober, David L. Buckeridge, Donald R. Olson

**Affiliations:** New York City Department of Health & Mental Hygiene; New York City Department of Health & Mental Hygiene; Public Health - Seattle & King County, WA; Virginia Department of Health; Georgia Division of Public Health; Georgia Division of Public Health; North Carolina Division of Public Health; NC-Detect, University of North Carolina, Chapel Hill, NC; Indiana Department of Health; Utah Department of Health; Utah Department of Health; Washington State Department of Health; Queen's University, Kingston, Ontario, Canada; University of Washington, Seattle, WA; University of Washington, Seattle, WA; McGill University, Montreal, Canada; International Society for Disease Surveillance; ^*^Research Director, International Society for Disease Surveillance; ^†^Director of Syndromic Surveillance, New York City Department of Health and Mental Hygiene, Queens, NY; ^‡^Associate Professor; Health Informatics and Global Health, Schools of Medicine, Nursing, and Public Health; University of Washington; ^§^Associate Professor, Department of Epidemiology and Biostatistics, McGill University, Montreal / Public Health Physician, Montreal Public Health and INSPQ and ^¶^International Society for Disease Surveillance (ISDS), Distributed Surveillance Taskforce

## Abstract

The Distributed Surveillance Taskforce for Real-time Influenza Burden Tracking and Evaluation (DiSTRIBuTE) project began as a pilot effort initiated by the International Society for Disease Surveillance (ISDS) in autumn 2006 to create a collaborative electronic emergency department (ED) syndromic influenza-like illness (ILI) surveillance network based on existing state and local systems and expertise. DiSTRIBuTE brought together health departments that were interested in: 1) sharing aggregate level data; 2) maintaining jurisdictional control; 3) minimizing barriers to participation; and 4) leveraging the flexibility of local systems to create a dynamic and collaborative surveillance network. This approach was in contrast to the prevailing paradigm for surveillance where record level information was collected, stored and analyzed centrally. The DiSTRIBuTE project was created with a distributed design, where individual level data remained local and only summarized, stratified counts were reported centrally, thus minimizing privacy risks. The project was responsive to federal mandates to improve integration of federal, state, and local biosurveillance capabilities. During the proof of concept phase, 2006 to 2009, ten jurisdictions from across North America sent ISDS on a daily to weekly basis year-round, aggregated data by day, stratified by local ILI syndrome, age-group and region. During this period, data from participating U.S. state or local health departments captured over 13% of all ED visits nationwide. The initiative focused on state and local health department trust, expertise, and control. Morbidity trends observed in DiSTRIBuTE were highly correlated with other influenza surveillance measures. With the emergence of novel A/H1N1 influenza in the spring of 2009, the project was used to support information sharing and ad hoc querying at the state and local level. In the fall of 2009, through a broadly collaborative effort, the project was expanded to enhance electronic ED surveillance nationwide.

## Introduction

The Distributed Surveillance Taskforce for Real-time Influenza Burden Tracking and Evaluation (DiSTRIBuTE) project is a case example for a new paradigm in the collection and sharing of public health data [1]. By connecting state and local jurisdictions that conducted electronic, emergency department (ED) syndromic surveillance, the DiSTRIBuTE project aimed to demonstrate the feasibility and utility of a fast, inexpensive, low burden model for population level respiratory, febrile and influenza-like morbidity surveillance. This effort emerged out of the unique collaborative environment of the International Society for Disease Surveillance (ISDS), where federal, state, and local public health agencies, academia, businesses, non-profit organizations, and other stakeholders leverage resources and technology to work together to advance disease surveillance practice and research.

The origin and evolution of DiSTRIBuTE was influenced by and responsive to the needs of public health departments and their use of syndromic surveillance systems for influenza monitoring in their own jurisdictions. The project was consistent with emerging models of population health data sharing and with changing federal perspectives on syndromic and biosurveillance architecture. Founded in this context, DiSTRIBuTE was based on a set of core principles: 1) share aggregate level data to minimize risk of exposure of personally identifiable information; 2) maintain jurisdictional control of surveillance data and information; 3) minimize barriers for health department participation; and 4) follow a collaborative approach to build on the flexibility of local systems and create a dynamic network. The resulting network was built upon the expertise and infrastructure of participating public health departments. 

In this paper, we set the context and describe the development of DiSTRIBuTE, presenting the goals and underlying principles behind the project and describing its evolution from the autumn of 2006 to the summer of 2009. Finally, we consider the lessons learned relevant to the use of syndromic surveillance for national influenza monitoring, and more generally to the sharing of population health data in the rapidly changing health information technology landscape.

## Background

Over the last decade, many public health agencies have implemented syndromic surveillance systems to provide early warning and detailed situational awareness of disease outbreaks, bioterrorist threats, and other ongoing health crises or events [2] [3] [4] [5]. These systems represent a potential innovation over other surveillance approaches due to their rapid collection of high volume, pre-diagnostic, electronic data, coupled with routine and automated application of detection algorithms and other analytic methods. The original rationale for implementing syndromic surveillance systems was to provide the earliest possible warning of unusual health events or emerging threats. Early detection can translate into rapid implementation of control strategies, and ultimately, mitigation of morbidity, mortality, economic loss, and threats to national security [2] [3] [4] [5]. These systems typically use existing electronic data sources and thus, compared to systems that require manual data collection, syndromic surveillance systems offer the potential for cost savings and more rapid collection of larger volumes of data.

Despite the potential advantages of these syndromic systems, they have not been embraced universally. This reticence has been due to concerns about the programs’ utility for initial outbreak and specific disease detection, limited public health funding and workforce resource constraints, particularly at the state and local level, and tensions between public health and national security priorities [2] [3] [4]. The need to invest in early detection of unusual events such as bioterrorism, pandemics, or other emerging health threats through syndromic surveillance systems has also been questioned in the context of the pressing need for public health departments to invest in existing surveillance systems aimed at monitoring notifiable diseases and outbreaks typically encountered in their jurisdictions. Public health departments that have adopted electronic syndromic surveillance systems, however, have reported that they increasingly use these systems to complement routine surveillance, most notably for influenza-like illness (ILI) syndromes, and for general large area morbidity trend monitoring [6].

As experience increased with the development and application of syndromic surveillance, evidence from health departments began to emerge that these data could provide important information at the local and regional levels to improve monitoring of seasonal and epidemic influenza [7] [8] [9] [10] [11]. This evidence led to public health interest in having more timely information about neighboring, regional, and national influenza trends. Users from health departments participating in the ISDS community felt that their state and local syndromic surveillance systems could provide rapid, representative, and accurate trends in their own jurisdictions. They also found it valuable to have information about surveillance trends in neighboring and "peer" health departments, and that sharing their information with other jurisdictions made those health departments in turn more willing to share their own surveillance information.

In the US, ILI surveillance has traditionally been conducted through a volunteer sentinel physician network by the Centers for Disease Control and Prevention (CDC) and coordinated through state and several large metropolitan health departments [12]. The CDC system, ILINet, monitors reported cases of ILI, defined clinically as individuals presenting with influenza, or fever with cough and/or sore-throat in the absence of another known cause. The ILINet cases are aggregated by week ending Saturday, typically reported to CDC during the following week manually via a web form, and presented as percent ILI to clinic visit ratios by region on the CDC website, typically by the end of that week. The system collects data nationwide, and includes a viral testing component whereby participating sentinel physicians submit clinical specimens to CDC periodically throughout the influenza season for laboratory testing.

The CDC ILI network is a highly valued public health surveillance system and serves the critical roles of monitoring ILI trends and collecting viral samples in all 50 states. Potential benefits of syndromic surveillance, however, over the traditional sentinel physician network include: faster provision of data due to electronic, automated data submission; lower burden on healthcare providers who would otherwise have to report manually; better stability of data, since providers might otherwise drop out or have delays if reporting manually; year-round reporting, unlike the sentinel system which only receives reports from the majority of participants during influenza season; availability of age-specific denominator data; flexible case definitions; and in many jurisdictions, potential for greater population coverage than with the sentinel physician network.

## The DiSTRIBuTE Project

### Models of Data Sharing

The DiSTRIBuTE effort was based on a practical philosophy of data sharing and on two key observations. First, for legal and organizational reasons, public health departments can share non-specific surveillance information and aggregate level data more easily than patient level records from health facilities within their jurisdictions. Second, public health departments are more willing to share information and data on disease patterns among trusted collaborators, particularly when only the minimum data needed to answer the public health questions are provided, and where jurisdictional control and authority over data are maintained. Previous national syndromic surveillance efforts, such as the original BioSense system, were top-down models which relied on centralized aggregation of detailed personally identifiable information, were not developed collaboratively and, consequently, were disconnected from the practical needs and resources of public health departments [13].

In 2006 and 2007, new federal level biosurveillance and pandemic preparedness recommendations, notably Homeland Security Presidential Directive, HSPD-21 [14] and the Pandemic and All-Hazards Preparedness Act, PAHPA [15], required that federal efforts be based on existing state and local biosurveillance and influenza surveillance systems. However, there were no clear guidelines regarding the coordination of these efforts, systems, and practices. During the same period, the Markle Connecting for Health Collaborative -- a public-private collaborative of over 100 health, policy, and technology leaders brought together by the Markle Foundation -- identified common challenges to data sharing in a wide range of population health efforts [16]. Many of these challenges were attributed to the current paradigm for analyzing population health data, which is typified by central collection of personally identifiable records, followed by data processing, cleaning and analysis. Notable problems with this model were a tendency to create data silos, lack of feedback to the original data holders, legal and practical restrictions to sharing individual-level data, delays in accessing or disseminating collected data, considerable cost to acquire data and concern over jurisdictional autonomy regarding use of health data and information. 

Drawing on experiences from multiple stakeholders, Markle Connecting for Health proposed principles intended to facilitate the sharing of population health data in support of effective decision-making [1]. These principles include collecting only summarized data with personally identifiable data being held at the source; cleaning and analyzing data at the source before sharing it in a standardized format; making aggregate data available across the network for analysis without requiring access to the original data; and building trust among entities in the network, enabled by having a set of policies and practices for jurisdictional control and data protection.

The DiSTRIBuTE project emerged out of the unique collaborative environment of ISDS, which fosters the creation of interdisciplinary, cross-agency collaborations that bridge research, practice and policy. The development of the project was also influenced by current thinking around models for sharing population health data, developing experience with syndromic surveillance for influenza, and the evolving national biosurveillance policy landscape in the United States.

### Project Goals

At the outset of the DiSTRIBuTE project, limitations in data sharing were evident in national influenza surveillance and biosurveillance practices, as reflected by position statements from within the public health community [13] [17] and Federal legislation and directives [14] [15]. Specific to ILI surveillance, limitations included: delays in reporting, high provider drop-out rates, burden on clinical and public health practice, limited flexibility with case definitions, lack of age-specific denominator, and lack of year-round reporting. Specific to biosurveillance practice, limitations included: creation of multiple separate data silos, lack of feedback to original data holders, legal and practical restrictions to sharing personal identifiable information, delays in accessing or disseminating collected data, considerable cost to acquire data, and concern over state and local jurisdictional autonomy. In an effort to address these limitations and needs, the ISDS DiSTRIBuTE project sought to develop a simple, low cost network for sharing aggregate data from ED syndromic surveillance systems that would protect privacy and allow jurisdictional data control. 

The primary goals of DiSTRIBuTE were to establish the feasibility of sharing aggregate population health data; and to assess the utility of regional and national sharing of ED syndromic surveillance data for influenza surveillance. These goals were also in line with the Markle Connecting for Health principles [16].

### Project Principles

In order to achieve the original goals of the project, a core set of principles were followed. These are summarized in Box 1, and discussed below.

Use Aggregate Data. Collection of aggregated data from health jurisdictions has advantages over individual-level raw data because it can sufficiently represent populations while protecting personally identifiable information. DiSTRIBuTE employed a simple data format that included an agreed upon minimum level of data detail for the epidemiological question and public health action of monitoring febrile, respiratory and ILI syndromes at the population level.

Maintain Jurisdictional Control. Public health departments are more likely to share surveillance data among trusted collaborators in an environment that protects jurisdictional control and where policies are defined in a participatory manner. The intent of this principle was to ensure that both the data framework and the exchange of surveillance findings and interpretation were suitably controlled by the participating jurisdictions.

Minimize Barriers to Participation. The use of a simple aggregate data format minimized one barrier to participation in the project.  Participants were initially asked to submit counts of cases measured according to the definitions and standards used in their existing systems. This flexibility lowered the barrier to entry into the project, deferred to state and local authority, and leveraged existing practices with local syndrome definitions and standards. This approach acknowledged the role of local context in extracting public health information from local clinical data. In other words, we assumed that local syndromes were defined based on regional variations in data collection standards, idiom, language, syndrome coding, hospital information systems, as well as other factors, and we wanted to build on this experience and expertise. 

Create a Collaborative Network. Data exchange and information sharing in the DiSTRIBuTE project was community-based, and participating jurisdictions were collaboratively involved in the development, specification, and implementation of the system, and in the data analysis and interpretation. The intent of this principle was to ensure that the data exchange framework was based on jurisdictional needs and priorities, and to foster data sharing among trusted collaborators in an environment where they defined the policies and controls in a participatory manner.

**Figure:**
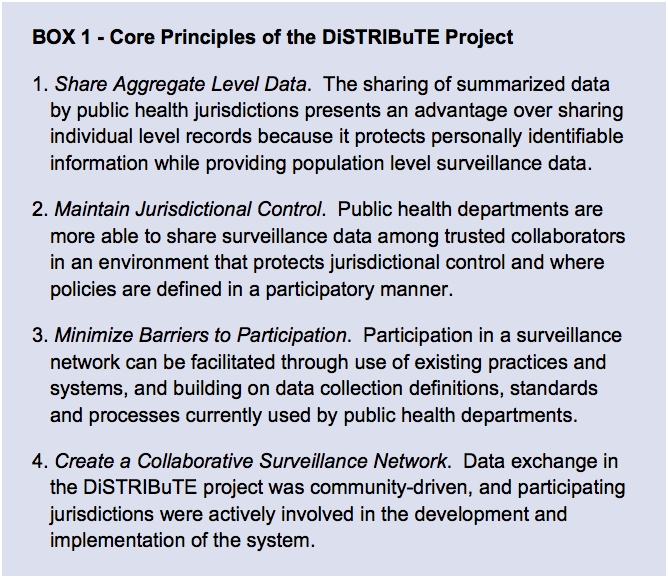

## The DiSTRIBuTE System

DiSTRIBuTE began with the comparison of syndromic surveillance trends between jurisdictions and with the sharing among syndromic surveillance practitioners of system coding and specifications. The public health utility of comparing influenza surveillance trends and the relative ease of sharing aggregate data, programming code, and system specifications between a small number of participant sites suggested that a framework and system could be created to scale the effort to a larger group of local, state, and international participating health departments.

Beginning in 2006, the DiSTRIBuTE project sought to enroll health departments that conducted ED-based syndromic influenza surveillance in their jurisdictions to voluntarily share their data [6] [7] [8] [9] [10] [11]. Many had expressed an interest in sharing data and collaborating in an effort to build a participatory, grassroots surveillance network since federal efforts at the time largely bypassed state and local systems, context, expertise and authority. 

Data characteristics and specifications in DiSTRIBuTE were originally aimed at capturing aggregate daily total and influenza-related syndrome counts of ED visits by predefined age-group and three-digit ZIP-code (ZIP-3). Participant sites reported aggregate counts by groups covering infants and toddlers (age <2 yrs), preschool-age (2-4 yrs), school-age (5-17 yrs), working-age adults in younger (18-44 yrs) and older (45-64 yrs) groups, and senior citizens (age 65+ yrs). Geographic information was originally requested to capture patients’ reporting ZIP-3, however, some participating sites chose instead to submit data based on ED facility ZIP-3, or to aggregate to larger regional areas than ZIP-3 (e.g., reporting data aggregated to the city or county level). The febrile, respiratory and influenza-like syndromes initially used in the project were requested to be comparable to a commonly used “fever/flu” syndrome that many participant sites were familiar with, and which many were currently using for surveillance in their jurisdictions [7].

### Syndrome Definitions

While participating sites generally did not use identical syndrome groupings to monitor influenza-related ED visits in their jurisdictions, the data were requested to be submitted as the preferred local syndrome grouping used by the health departments, with the intent of representing the patterns and trends that the jurisdiction wished to have shown for their region. During the proof of concept period, as sites shared data and observed each other’s trends, there was interest in comparing syndromes based on concepts rather than strictly on defined coding standards [18]. There also was interest in applying more broad and sensitive febrile and respiratory influenza-related groups, and more narrow and specific influenza-like syndromes that were more closely analogous to the traditional clinical surveillance definition of ILI (a presentation with influenza, or fever with cough and/or sore throat) [11] [12].

In a pilot comparison of two DiSTRIBuTE jurisdictions, each used the other’s syndrome coding and applied it to their own data to compare the resulting surveillance trends with the syndromes they used locally. The results suggested that using the locally defined syndromes created surveillance time-series that better matched the viral isolate data of confirmed influenza cases locally [19] [20]. As the project progressed, participating sites began to reevaluate their own syndrome definitions, and in many cases began to move toward common syndrome concepts through stepwise adaptations of local syndrome definitions, coding, and preferences. As sites looked at each other’s data more, they better understood the heterogeneity of the syndrome groups. And when they assessed their own syndrome characteristics, many reported making minor coding changes that were consistent with making the syndrome concepts and definitions closer and more comparable. This followed a similar process to standardization in other information technology efforts, where standards were aligned with the measurable and practical needs and interests of the users – and standards became “standard” because they became the normal case in the field [21].

During the proof of concept period, work focused on comparison of influenza surveillance systems, evaluation of the model, and harmonization of common ILI syndromes [19] [20] [22] [23]. Similarly, acute gastroenteritis syndrome indicators were implemented in the project, with the goal of being able to monitor winter-seasonal increases believed due primarily to norovirus and rotavirus epidemics across the DiSTRIBuTE network. Implementing and sharing acute gastroenteritis trends also served to assess to what degree the DiSTRIBuTE model could be generalized from monitoring population level ILI morbidity trends to monitoring trends in another constellation of syndrome groups believed closely tied to two recurring winter-seasonal epidemic viral diseases. The implementation of these new groups required participants to adopt or modify syndromic output to include an additional column of data and report historical baselines of the new syndrome groups. Additionally, during the proof of concept period, evaluation efforts and informal *ad hoc* comparisons were done within the network, notably with respect to an assessment of DiSTRIBuTE data with Google Flu Trends [23] and with the emergence of novel A/H1N1 influenza in the spring of 2009. 

### Technical Infrastructure

The technical infrastructure to initially support DiSTRIBuTE employed an incremental approach to automating and securing the manual processes initially used in the pilot, and then by early participants. Data were originally exchanged by email attachments using comma separated value (CSV) files in similar formats. An automated system was implemented to permit secure file transmission using the secure shell file transfer protocol (SFTP) and utilizing existing commercial software at the health jurisdiction, or equivalent free software identified by the project. The users sent their data files to a central server where automated software services received the data files, performed basic error detection (including data type errors, simple range checking, and improper coded values), and transformed the data into a common format (allowing for site-specific variation in the composition and layout of the CSV file). The resulting files shared a common syntax that were free of both format and simple content errors. Examples of the latter included violations of simple data type, basic range checking of results, and constrained enumerated values (such as age ranges for age stratification), and were free of format and simple content errors, including violations of simple data type. The standardized files were parsed and their content imported into a simple database, which was implemented using the open source MySQL database package. Open source software was employed for the entire centralized server, using the "Linux, Apache, MySQL, PHP, Perl" development framework.

### Visualization of Data

The display of DiSTRIBuTE trends was created as the primary means for sharing and presenting data from participating health jurisdictions. Initially, weekly aggregate ratios of febrile respiratory or influenza-like syndromes, based on each participating site’s routine syndromic criteria for monitoring seasonal influenza, to total ED visits, were visualized as regional weekly time-series (Figure 1), and as age-specific temporal epidemic response surface (TERS) plots (Figure 2). The regional time-series plots presented variation in relative baseline levels during non-influenza periods during the proof of concept period, from lower quartile weekly ratios of 1.4% to over 6% of total ED visits. Peak seasonal epidemic influenza levels by participating health departments, ranged from 6% to over 16%. The variation in non-influenza period baseline levels and peak seasonal epidemic levels indicated that the overall magnitude of the data were not directly comparable. However, the relative pattern of the time-series reported by participating systems were noted as being consistent with regional, state and local influenza surveillance systems and measures [6] [7] [8] [9] [10] [11]. 

While participating DiSTRIBuTE sites were not representative of the whole nation on a population basis, taken as an aggregate 13% convenience sample of the U.S., the data for the 2006-2007 and 2007-2008 influenza seasons were highly correlated with national ILI surveillance data [22] [23] (Figure 1). 

**Figure:**
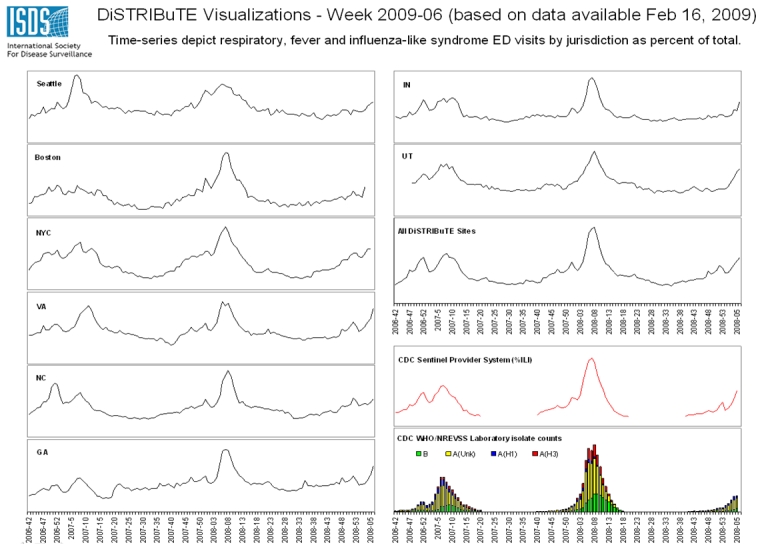

**FIGURE 1** - DiSTRIBuTE time-series visualizations by jurisdiction, February 16, 2009. ILI syndrome time-series are plotted as ratios for DiSTRIBuTE and national CDC sentinel ILI, and as counts by subtype for viral influenza isolate data [12]. DiSTRIBuTE data were typically reported one or more weeks ahead of sentinel reporting data. Pearson correlation between all DiSTRIBuTE sites and CDC ILI for the 2006-2007 and 2007-2008 influenza seasons was 0.96 (p<0.01). 

**Figure:**
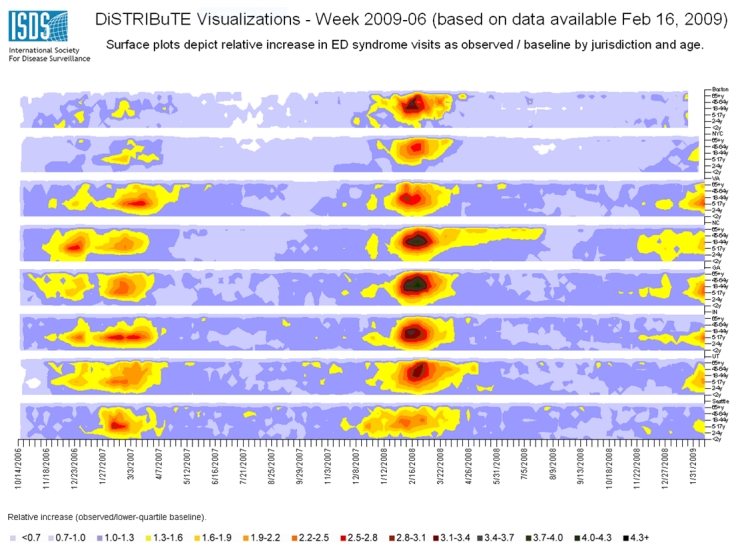

**FIGURE 2** - DiSTRIBuTE age-specific visualization, February 16, 2009. ILI syndrome time-series as age-specific temporal epidemic response surface (TERS) plots for 2006-2009 are shown as relative increase, calculated as observed over the lower-quartile baseline ratio by age-group and jurisdiction [11]. Age-groups are stratified into ranges representing infants and toddlers (age <2 yrs), preschool-age (2-4 yrs), school-age (5-17 yrs), younger adults (18-44 yrs) older adults (45-64 yrs), and senior citizens (age 65+ yrs).

The age-specific visualizations presented participant jurisdiction data as an interpolated surface gradient of the relative magnitude of visits, calculated as observed ratios over lower-quartile baseline, by age-group through time [11]. The plots presented a snapshot of age-specific trends and intensity by jurisdiction, with notable characteristics such as the age-specific timing and relative magnitude of the predominant circulating epidemic viruses in a particular jurisdiction (Figure 2). 

National surveillance trends in ILINet from 2008 and later were highly correlated with DiSTRIBuTE data, however ILINet included three DiSTRIBuTE participating health department ED systems during this period. The inclusion of these three state and local syndromic systems represented a large portion (roughly 27%) of the total national ILINet visit volume, and this resulting overlap prevents direct comparison of the systems without disaggregating the regional data. A suitable gold standard for evaluation of population level influenza trends, whether from clinical sentinels or syndromic ED data, is time-series of laboratory confirmed influenza infections. The national viral isolate data, as a proportion of weekly viral tests positive for influenza, were highly correlated with the combined DiSTRIBuTE data (Figure 3).

**Figure:**
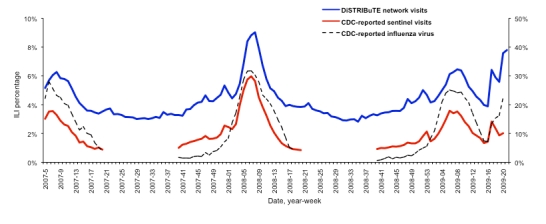

**FIGURE 3** - DiSTRIBuTE and CDC ILINet and viral surveillance data, 2007-2009. DiSTRIBuTE ILI syndrome time-series are shown as ratios, with national CDC sentinel ILI and viral influenza isolate data [12]. Pearson correlation between all DiSTRIBuTE sites and CDC viral surveillance were significant for the 2007-2008 (0.90, p<0.01) and 2008-2009 (0.86, p<0.01) influenza seasons.

Originally, visualizations and findings were summarized and disseminated via email, on the ISDS website www.syndromic.org, and through meetings, webinars and presentations. Later visualizations were presented on a user website hosted at the University of Washington, where a series of automated queries and statistical and graphing routines were implemented as scripts in the R statistical programming language and run on a daily basis to produce images for both public and restricted-access web applications. The public site showed time series of daily and weekly syndrome ratios. The restricted site was accessible by only those jurisdictions contributing data to DiSTRIBuTE (Figure 4). It included both daily and weekly ratios for several ILI and gastroenteritis syndromes, as well as their constituent data, syndrome counts and visit totals.

As the number of participant sites grew, features were added to enhance the ability to create custom visualizations, as well as to enter descriptive data, or metadata, specific to each site. These data included information needed to manage the creation of combined or composite time series, control the flow of data based on specific terms of participation for particular sites, specify order of appearance for menu items and visualizations, provide denominator information such as estimates of the population covered or number of hospitals in a particular jurisdiction, and contact information for epidemiologists and IT staff with each jurisdiction and site. 

**Figure:**
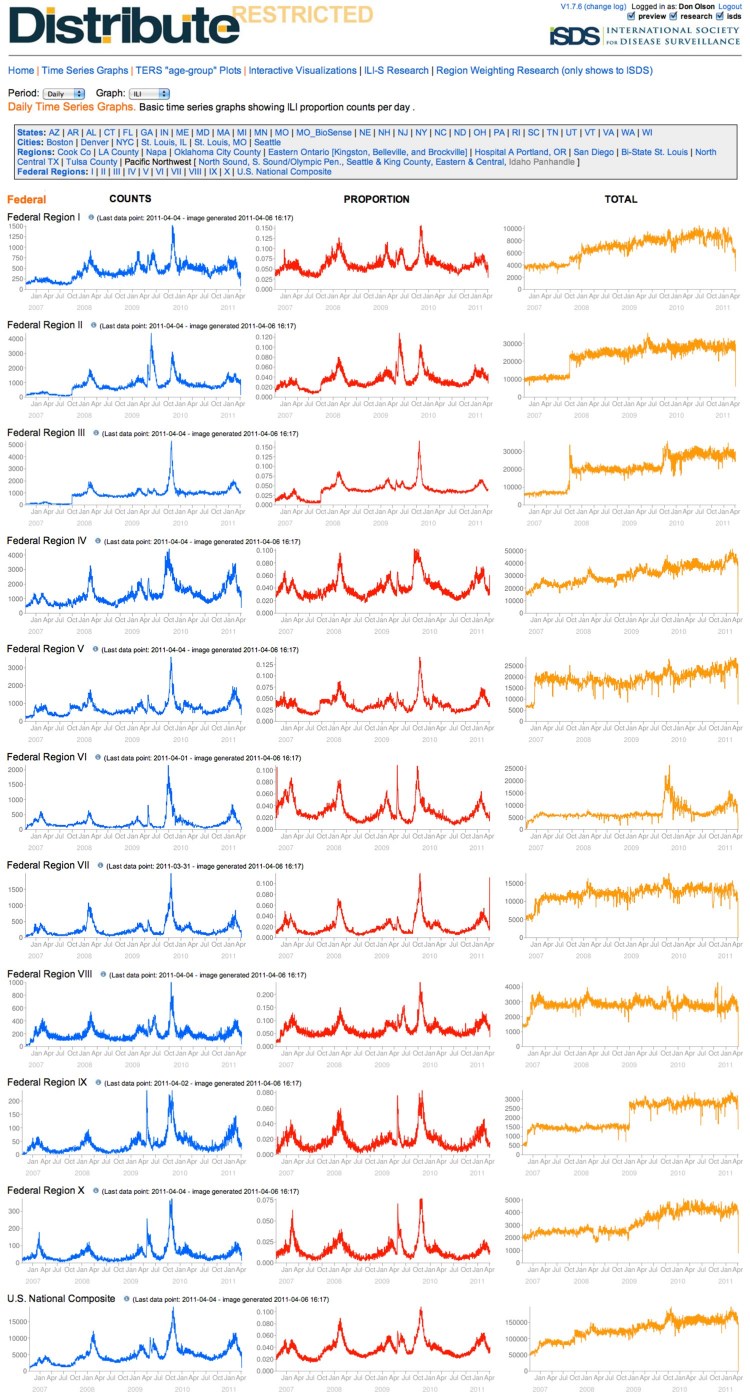

FIGURE 4 - National and HHS Regional DiSTRIBuTE time-series, 2007-2011. Daily trend data are shown for ILI counts, ILI ratios and total ED visits as presented on the ISDS restricted website (Accessed April 6, 2011). The 42 DiSTRIBuTE participating state, county and city jurisdictions are aggregated by federal surveillance region: Region 1, Boston, Connecticut, Maine, Massachusetts, New Hampshire, Rhode Island, Vermont; Region 2, New Jersey, New York City, New York State; Region 3, Maryland, Pennsylvania, Virginia; Region 4, Florida, Georgia, North Carolina, South Carolina, Tennessee; Region 5, Cook County Illinois, Indiana, Michigan, Minnesota, Ohio, St. Louis Illinois, Wisconsin; Region 6, Arkansas, North Central Texas, Oklahoma City and Tulsa Counties Oklahoma; Region 7, Missouri, Nebraska; Region 8, Denver Colorado, North Dakota, Utah; Region 9, Arizona, Los Angeles, Napa and San Diego Counties California; Region 10, Portland Oregon, Seattle and King County and Washington State.

### The Pandemic Phase

The development and evolution of the DiSTRIBuTE project changed dramatically with the emergence of novel A/H1N1 influenza in April of 2009. With the first influenza pandemic in over 40 years, the demands on local, state, and national public health practice increased. There were notable lessons learned with the DiSTRIBuTE effort during the emergence and progression of the pandemic in the spring of 2009 and with the public health concern over its anticipated recrudescence the following summer and fall – these lessons are presented in the sections below. The DiSTRIBuTE findings and the federal surveillance needs in response to the pandemic [24] led to the implementation and expansion of DiSTRIBuTE nationwide through a collaborative effort with the Centers for Disease the Centers Disease and Prevention (CDC), as part of an existing cooperative agreement with the Public Health Informatics Institute (PHII).

### Feasibility of the Data Sharing Model

The DiSTRIBuTE project demonstrated that it was feasible to share population health ILI data nationally in a manner that addresses the needs of local jurisdictions [13] [17] and is consistent with the Markle Connecting for Health principles [16]. Although it was clear that submitting aggregate data, as opposed to individual records, encouraged data sharing among participants, it did not altogether eliminate concerns about control and use, particularly with regard to sharing provisional and near real time data.

The principle of keeping barriers to participation low facilitated the growth of the DiSTRIBuTE network. One of the other apparent effects of this principle was that data submitted by different regions often reflected different syndromic definitions, with varying sensitivity and specificity for monitoring conditions of interest. Some epidemiologists and other observers perceived this difference as a limitation to overall data quality, particularly with regard to comparing data .across regions. However, others viewed the heterogeneity as something that was recognized and accounted for by the jurisdictions, where definitions were derived empirically or in response to a local surveillance need. Recognition of these differing perspectives led almost immediately to ongoing collaborative assessment of syndrome definitions and other surveillance practices.

### Applying the Model

The success of the DiSTRIBuTE project in demonstrating the feasibility of implementing an innovative low cost model for national syndromic influenza surveillance suggests that it is worth considering expansion of this model to other surveillance activities and to other fields in public health and health care. In DiSTRIBuTE, the data collection process was generalized to acute gastroenteritis syndromes, and potential expansion, for instance, to the monitoring of quality of care would be more ambitious and rewarding. In fact, elements of the DiSTRIBuTE model could conceivably be applied to any system that relies on collaborative networks, rigorous data collection, detailed and comprehensive data, and ongoing technical support.

The lessons regarding standardization of influenza surveillance are also instructive. The issue of standardization remains a major challenge for syndromic surveillance. While relative disease patterns and trends can be compared between systems with heterogeneous syndrome definitions, the ability to compare more direct epidemiological measures is limited. ISDS and collaborators continue to work on strategies to find common ground for syndrome definitions and coding that can inform national agendas for syndromic surveillance.

Providing an emphasis on establishing data transmission while accepting variations in data standards and then allowing the community to move to collaborative harmonization appears to be a viable approach and one that offers an alternative to top-down standards-setting approaches, which can be slow to complete and result in high technical barriers to participation. It also needs to be acknowledged that population surveillance of aggregate data has inherent limitations for queries and analysis at deeper levels of detail.

The question of cost is also important to consider. The DiSTRIBuTE model demonstrated that a national network can be organized to support existing state and local systems, expertise and infrastructure for both public health surveillance and emergency preparedness and response with modest funding (i.e., less than $200,000 per year during the proof of concept period, 2006 to 2009). For the contributing systems, the necessary costs incurred by state and local jurisdictions to support syndromic surveillance were offset in part or in total through federal funding by the CDC through Public Health Emergency Preparedness (PHEP) cooperative agreements, and Public Health Emergency Response (PHER) grants, and through foundation support or direct spending by the jurisdictions themselves. While the total costs required for developing and operating the existing infrastructure of state and local syndromic surveillance through federal PHEP and PHER funding is unknown, federal level efforts to create a national centralized network, such as BioSense, cost roughly $30 million per year during the period 2003 to 2009 [25] [26]. 

## Conclusion

During the period from 2008 to 2009, the participating DiSTRIBuTE jurisdictions represented over 16% of the US population, and captured roughly 13% of all ED visits nationwide. By early 2011, the network had grown to over 43 reporting sites and captures over 40% of all ED visits. The DiSTRIBuTE project has changed the practice of syndromic surveillance in the US. It promoted an assessment of practice patterns and helped to identify variations among different public health jurisdictions, while offering national perspective and peer comparisons. DiSTRIBuTE has been useful for understanding the current landscape of syndromic  surveillance, enhancing data quality, and creating a framework that can be applied to syndromes beyond influenza.

In 2009, DiSTRIBuTE was identified as a case example in a White House recommendation by the Presidents Council of Advisors on Science and Technology for implementation of a nationwide ED surveillance network as part of the preparedness and response effort to the 2009 influenza A/H1N1 pandemic [24]. The project was highlighted in Senate testimony by the White House Chief Technology Officer as an example of the federal ‘Open Government Directive’ in moving public health surveillance research into development and deployment, most notably for it’s grass-roots participation, low cost to acquire data and unprecedented public transparency [27]. DiSTRIBuTE has also impacted the way national health information technology initiatives view syndromic surveillance [1].  In 2010, the DiSTRIBuTE community initiated and participated in a process led by ISDS to define business standards for syndromic surveillance and create messaging standards in support of “Meaningful Use” certification (Recommendation: Core Processes and EHR Requirements for Public Health Syndromic Surveillance, available at www.syndromic.org).

The significant progress in population level syndromic surveillance that the DiSTRIBuTE project made during the proof of concept period from 2006 to 2009 demonstrated that a national network can be created with modest funding and without unnecessary exposure of record level information or unnecessary burden on health practitioners – and all while supporting existing state and local public health systems and capacity and providing rapid, high volume, summary electronic surveillance. Electronic data processes and approaches like this can not replace clinician reporting or laboratory testing of clinical samples collected by sentinel physicians, but they can augment these systems in a way that confirms and expands our understanding of population level disease trends. Tremendous potential remains for DiSTRIBuTE to continue to improve the nation’s ability to prepare for, monitor and respond to disease outbreaks.

## Acknowledgements

The authors wish to acknowledge the initiation, insight, guidance, and support on this project from founding ISDS President Farzad Mostashari. During the proof of concept period, the ISDS Board of Directors provided institutional oversight and guidance (2006 to 2010): John S. Brownstein, David L Buckeridge, Howard S. Burkom, Jean-Paul Chretian, Duncan Cooper, Julia Gunn, Lori Hutwagner, William B. Lober, Joe Lombardo, Farzad Mostashari, Marc Paladini, Julie Pavlin. The authors thank Laura Streichert for assistance with editing the manuscript.

Participants in the ISDS DiSTRIBuTE Working Group meetings provided critical guidance and content expertise during the project, from proof of concept to nationwide implementation. Participants and data contributing sites included the following: New York City Department of Health & Mental Hygiene, Marc Paladini, Don Olson, Richard Heffernan; Boston Public Health Commission, Justin Pendarvis, Julia Gunn; Public Health - Seattle & King County, Atar Baer; Virginia Department of Health, Michael A. Coletta; Georgia Division of Public Health, Karl Soetebier, Erin L. Murray; North Carolina Division of Public Health, Lana Deyneka, Amy Ising, Jeffrey Engels; Indiana Department of Health, Ryan Gentry, Michael Wade; Utah Department of Health, Melissa Dimond, Felicia Alvarez, Robert T. Rolfs; Washington State Department of Health, Bryant Thomas Karras; Kingston, Ontario, Canada, Kieran Moore. Working Group participants with collaborating academic or non-profit groups: University of Washington, William B Lober, Debra Revere, Ian Painter; Johns Hopkins, Howard S. Burkom; Markle Foundation, Jill Schulmann, Carol Diamond. Participants with data submission in-progress or ad-hoc during the proof of concept period, 2006 to 2009: New York State Department of Health, Geraldine Johnson, Hwa-Gan Chang; Maine Department of Health, Amy Robbins; Vermont Department of Health, Pam Berenbaum; Duval County, Florida, Taj Azarian, Saad Zahir; Houston, Patricia Sinawe; Cook County, Illinois, Megan Patel; North Dakota Department of Health, Julie Wagendorf, Lindsey Vander Busch; Wisconsin State Department of Health, Richard Heffernan; Connecticut State Department of Health, Alan Siniscalchi, Katherine Purviance; Florida State Health Department, Aaron-Kite Powell; Ohio Department of Health, Brian Fowler, Richard Thomas; INSERM and UMPC, Paris, France, Camille Pelat, Alain-Jacques Valleron.

During the H1N1 pandemic, the US CDC played a vital role in the expansion of Distribute from a proof of concept project to a nationwide project that currently includes forty-two participating public health jurisdictions. Beginning in August 2009, the group that is now the Public Health Surveillance and Practice Office (PHSPO) of the CDC provided, through the BioSense program and other mechanisms, substantial support to the Distribute project in terms of funding; allocation of personnel; site outreach, recruitment and technical assistance; and coordination with other groups at CDC, including the Influenza Division. As of August 2011, more than one third of the jurisdictions participating in Distribute submit their data via CDC (either through BioSense or through reporting data directly to CDC). In summary, the CDC provided support that was necessary for the rapid expansion of Distribute in the Fall of 2009, and the CDC continues to support and fund the ISDS Distribute project and its community of practice.

The participating local and state public health agencies played a vital role in Distribute before and during the pandemic period. Through their voluntary and dedicated participation, the system rapidly expanded in 2009 from 10 to 35 contributing state and local health jurisdictions. Many of the approaches to improving data quality, analysis, and visualization were initiated by staff from these agencies, who devoted significant time and energy, as well as local IT resources, to ensuring that Distribute could expand to provide National coverage during a public health emergency.

## Funding Information

The development and proof of concept phase of the ISDS DiSTRIBuTE project was supported by the Centers for Disease Control and Prevention (CDC) through a cooperative agreement with the National Association of County and City Health Officials (NACCHO), 2006 to 2008, and the Markle Foundation, 2008 to 2009 (grant #081005BP-Q). Implementation and expansion of DiSTRIBuTE into a nationwide system was supported by the Markle Foundation, 2009 to 2010 (grant #101003BP-B), and by the CDC through a cooperative agreement with the Public Health Informatics Institute (PHII), 2009 to 2011.

## Author Contributions

ICMJE criteria for authorship read and met, DRO, MP, WBL, DLB; agree with the manuscript’s results and conclusions, DRO, MP, WBL, DLB; wrote the first draft of the paper, DRO and MP; revised the paper for substantive content and interpretation, DRO, MP, WBL, DLB. Contributed to the roundtable meetings and discussions that contributed to the development of the project, led to the paper, and were provided opportunity to review and revise paper, ISDS DiSTRIBuTE Working Group members.

## Competing Interests

The authors have declared that no competing interests exist. 
